# Single transcranial direct current stimulation in schizophrenia: Randomized, cross-over study of neurocognition, social cognition, ERPs, and side effects

**DOI:** 10.1371/journal.pone.0197023

**Published:** 2018-05-07

**Authors:** Yuri Rassovsky, Walter Dunn, Jonathan K. Wynn, Allan D. Wu, Marco Iacoboni, Gerhard Hellemann, Michael F. Green

**Affiliations:** 1 Department of Psychology, Bar-Ilan University, Ramat-Gan, Israel; 2 Gonda Multidisciplinary Brain Research Center, Bar-Ilan University, Ramat-Gan, Israel; 3 Department of Psychiatry and Biobehavioral Sciences, UCLA Semel Institute for Neuroscience and Human Behavior, Los Angeles, CA, United States of America; 4 Department of Veteran Affairs VISN-22 Mental Illness Research, Education and Clinical Center, Los Angeles, CA, United States of America; 5 Department of Neurology, University of California, Los Angeles, California, United States of America; 6 Ahmanson-Lovelace Brain Mapping Center, University of California, Los Angeles, California, United States of America; Maastricht Universitair Medisch Centrum+, NETHERLANDS

## Abstract

Over the last decades, the treatment of schizophrenia has shifted fundamentally from a focus on symptom reduction to a focus on recovery and improving aspects of functioning. In this study, we examined the effect of transcranial direct current stimulation (tDCS) on social cognitive and nonsocial neurocognitive functions, as well as on electroencephalogram (EEG) measures, in individuals with schizophrenia. Thirty-seven individuals with schizophrenia were administered one of three different tDCS conditions (cathodal, anodal, and sham) per visit over the course of three visits, with approximately one week between each visit. Order of conditions was randomized and counterbalanced across subjects. For the active conditions, the electrode was placed over the left dorsolateral prefrontal cortex with the reference electrode over right supraorbital cortex. Current intensity was 2 mA and was maintained for two 20-minute sessions, with a one hour break between the sessions. Assessments were conducted immediately following each session, in a counterbalanced order of administration. No systematic effects were found across the social and nonsocial cognitive domains, and no significant effects were detected on event-related potentials (ERPs). The very small effect sizes, further validated by *post-hoc* power analyses (large Critical Ns), demonstrated that these findings were not due to lack of statistical power. Except for mild local discomfort, no significant side effects were reported. Findings demonstrate the safety and ease of administration of this procedure, but suggest that a single dose of tDCS over these areas does not yield a therapeutic effect on cognition in schizophrenia.

**Trial registration:** ClinicalTrials.gov NCT02539797.

## Introduction

Considerable evidence indicates that cognitive factors are key determinants of functional outcome for schizophrenia [1–3]. These cognitive factors can be divided into neurocognition and social cognition. Neurocognition refers to a wide range of performance abilities in domains such as learning and memory, vigilance / attention, speed of processing, reasoning and problem solving, and working memory [4, 5]. Social cognition refers to cognitive abilities that are needed to perceive, interpret, and process social information and include domains such as emotion perception, social context processing, attributional bias, and theory of mind [6, 7]. Both neurocognitive and social cognitive deficits have been extensively documented in schizophrenia literature and are considered a core feature of the illness [8–10].

In addition to the considerable efforts in recent years to stimulate the development of cognition-enhancing drugs [11, 12], other approaches to enhance patients’ cognitive functions have been explored. Neurostimulation has advantages over pharmacological therapy in reducing many potentially dangerous systemic side effects. Currently transcranial direct current stimulation (tDCS) has been developed as non-invasive tool using neurostimulation to treat epilepsy [13]. Unlike other non-invasive brain stimulation techniques, such as transcranial electrical stimulation or transcranial magnetic stimulation, tDCS does not induce neuronal firing by suprathreshold neuronal membrane depolarization but rather modulates spontaneous neuronal network activity [14]. In a review of 567 tDCS sessions administered over cortical areas on a total of 102 healthy participants and patients, none requested the stimulation to be terminated or needed any medical intervention during or after tDCS administration [15].

Despite the safety and tolerability of this procedure, findings regarding its potential clinical benefits in schizophrenia have been mixed. Early studies of single tDCS administration in schizophrenia reported positive effects [16–18]. For example, Brunelin et al. (2012) reported preliminary data showing that bifocal stimulation (anodal over DLPFC and cathodal over tempo-parietal cortex) improved scores on measures of auditory hallucinations and overall positive and negative symptoms in 2 patients with refractory schizophrenia. The clinical efficacy appeared immediately after stimulation sessions and was maintained or continued to improve during at least 3 months. The authors noted that no adverse events were reported, and that the two patients only described a transient mild tingling or a slight itching sensation associated with the onset of stimulation, thereby confirming the safety of this technique [16]. Additionally, Vercammen et al. (2011) examined whether anodal tDCS to the left DLPFC would reverse probabilistic association learning deficits in 20 individuals with schizophrenia. The authors reported no adverse effects following the procedure and demonstrated a beneficial effect of tDCS on performance, as assessed by the weather prediction test, in a subsets of individuals [19].

Other studies, however, reported negative findings. For example, in two randomized, double-blind, controlled trials, Fitzerald et al. (2014), examined the effects of unilateral and bilateral tDCS (anodal stimulation to the prefrontal cortex and cathodal stimulation to the temporoparietal junction) in patients with persistent hallucinations and negative symptoms of schizophrenia. They reported that, despite the tolerability of the procedure, neither unilateral nor bilateral tDCS resulted in a substantial change in either hallucinations or negative symptoms [20]. Mattai et al. (2011) investigated the tolerability aspects of tDCS in childhood-onset schizophrenia. The investigators administered bilateral anodal DLPFC stimulation or bilateral cathodal superior temporal gyrus (STG) stimulation to children between the ages 10–17. While demonstrating the safety and tolerability of this procedure in a pediatric population, no changes in cognitive measures in favor of any stimulation condition were observed [21].

The present study was an effort to evaluate the effect of single tDCS administration in schizophrenia on a broad range of social cognitive, nonsocial neurocognitive, and EEG measures. We employed a commonly used montage in cognitive enhancement tDCS studies over the course of three sessions, with approximately one week between each session: anodal tDCS over left DLPFC (cathodal over right supraorbital), cathodal tDCS over left DLPFC (anodal over right supraorbital), and sham stimulation. Using a reasonably well-powered cross-over design, we sought to detect a differential effect of these types of stimulation on performance-based tasks and brainwave activity that are mediated by frontal brain regions.

## Material and methods

### Participants

Participant recruitment and follow-up were conducted between May 2015 and July 2016. As described in detail previously [15, 17], participants were recruited by placing ads and receiving referrals from treating clinicians at outpatient clinics at the UCLA and the VA Greater Los Angeles Healthcare System and through presentations in the community. All patients were administered the Structured Clinical Interview for DSM-IV (SCID-P) [22]. All interviewers were trained to administer the SCID by the Diagnostic Core of the Mental Illness Research, Education, and Clinical Center (MIRECC) Treatment Unit, and were required to obtain a Kappa of 0.75 for key psychotic and mood items before proceeding to interview participants independently. Participants were included if they met the DSM-IV diagnostic criteria for Schizophrenia or Schizoaffective Disorder [23], were between the ages of 18–55, were able to understand spoken English sufficiently to comprehend testing procedures, and were under the current ongoing care of a psychiatrist, psychologist, or other qualified mental health professional. Patients were excluded if they had an identifiable neurological condition, metal in the cranium, intracardiac lines, cardiac pacemaker, medication pump, or increased intracranial pressure, psychiatric inpatient hospitalization in the last three months, substantial changes in their antipsychotic medications during the previous 6 weeks, IQ < 70 based on reading ability, or met criteria for substance dependence in the last six months.

**Fig 1:**
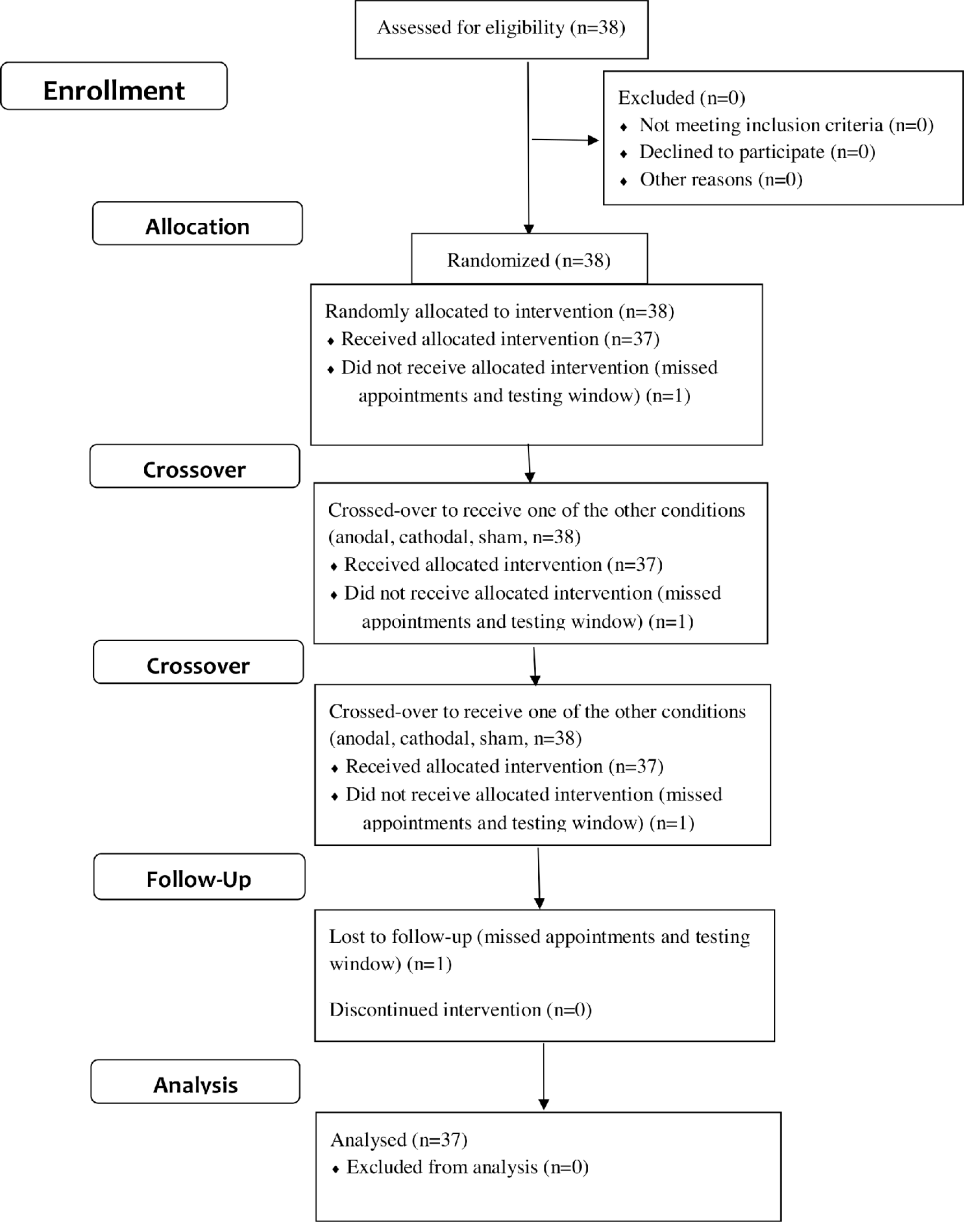
CONSORT flow chart.

The present study included 38 individuals (one participant was lost to follow-up after missing several appointments and testing window; see Fig 1) with schizophrenia or schizoaffective disorder (68% males), all receiving antipsychotic medications (93% atypical). Mean age was 42.7 (SD = 8.57; range = 23–55), mean participants’ education was 12.8 (SD = 2.11; range = 8–18), and mean paternal education was 13.6 (SD = 3.51; range = 3–20). Patients’ mean illness chronicity was 20.1 years (SD = 9.54; range = 3–43). Mean symptom ratings on the Brief Psychiatric Rating Scale (BPRS) was 42.1 (SD = 12.0; range = 25–77) and on the Scale for the Assessment of Negative Symptoms (SANS) was 41.0 (SD = 16.0; range = 13–80). All participants gave written informed consent after receiving a full explanation of the research according to procedures approved by the UCLA and the VA Greater Los Angeles Healthcare System Institutional Review Boards. The approved protocol is included in supporting information (see S1 Protocol). The trial was registered at www.ClinicalTrials.gov (NCT02539797). The authors confirm that all ongoing and related trials for this intervention are registered.

### Assessments

Symptom ratings were collected using the expanded BPRS [24] and the SANS [25]. Standardized social cognitive and nonsocial cognitive assessments included a number of primary measures that have been used in schizophrenia research [7]. Participants also rated on a 5-point Likert scale (ranging from none to severe; 0–4) common side effects that are typically reported following tDCS administration [15, 17]. The measures administered are listed in Table 1 and are described fully below.

**Table 1:** Measure administered to assess the effect of stimulation.

**Social Cognitive Measures**
MSCEIT
EIT
TASIT
EAT
**Nonsocial Cognitive Measures**
MCCB
**EEG Measures**
MMN
P300
N170

MSCEIT: Mayer-Salovey-Caruso Emotional Intelligence Test; EIT: Emotion Identification Test; TASIT: The Awareness of Social Inference Test; EAT: Emphatic Accuracy Task; MCCB: MATRICS Consensus Cognitive Battery; EEG: Electroencephalogram; MMN: Mismatch Negativity; The order of assessments was fully counter-balanced across subjects.

#### Social cognitive measures

Managing Emotions component of Mayer-Salovey-Caruso Emotional Intelligence Test (MSCEIT) [26] consists of 141 items and 8 ability subtests, which assess four components of emotional processing. In this study, only branches 1 and 4 were administered, focusing on the Managing Emotions component. Emotion Identification Test (EIT) from the Ekman Program is a test of identification of facial emotion based on photographs from the software developed by Ekman [27]. It includes 56 color photographs of eight different individuals displaying facial expressions of six basic emotions (happy, sad, angry, afraid, surprised, and disgusted). The Awareness of Social Inference Test (TASIT) [28] consists of 16 videotaped scenes with two or three actors appearing in each scene. The scenes involve two types of conversational exchanges, enacted as either a lie (a white lie or a sympathetic one) or sarcasm. After each scene, participants answer questions (yes/no) about the actors’ communicative intentions, such as whether the actors wanted the literal or nonliteral meaning of their messages to be believed. The Empathic Accuracy Task (EAT) [29] assesses the accuracy of empathic judgments using 12 video clips (six positive and six negative events), in which individuals (referred to as “targets”) discuss a positive or negative autobiographical event. As participants watch the clip, they use a 9-point scale to continuously rate how positive or negative they believe the target was feeling at each moment.

#### Nonsocial neurocognitive measures

The neurocognitive assessment consisted of the MATRICS Consensus Cognitive Battery (MCCB), which assesses seven separate cognitive subdomains, including one measure of social cognition, the managing emotions component of the MSCEIT [4, 30, 31]. Given the diminishing tDCS effect over time [32], we included four nonsocial neurocognitive tasks: speed of processing, working memory, verbal memory, and reasoning/problem solving. Normed T scores were calculated for each cognitive subdomain, as well as the cognitive composite score consisting of the average across the four subdomains.

#### EEG measures

EEG was acquired with a Neuroscan NuAmps amplifier (Compumedics USA, El Paso, Texas) continuously throughout the session. Data were sampled at 1000 Hz from DC to 100 Hz. Nineteen cap-mounted Ag-AgCl electrodes were positioned using a modified international 10–20 system placement scheme. Additionally, 2 electrodes were used to measure vertical electro-oculogram (placed above and below the left eye) and used in eyeblink correction [33]. All electrodes were referenced to the mastoids, unless otherwise noted.

Mismatch negativity (MMN) was measured using a passive attention auditory duration deviant paradigm. Subjects were presented with binaural tones (1 kHz 85 dB sound pressure level, with 10 ms rise/fall). Standard (90% probability; 50 ms duration) and duration-deviant (10% probability; 100 ms duration) tones were presented in a fixed, pseudorandom order. Full details regarding stimulus presentation and EEG recording and analysis are presented elsewhere [34]. The mean amplitude between 135–205 ms of the deviant-standard difference wave was calculated at electrode Fz.

Following MMN, P300 wave was measured using an active attention auditory oddball paradigm. Subjects were presented with standard (88% probability; 1 kHz frequency; 100 ms duration) and target (12% probability; 1.5 kHz frequency; 100 ms duration) tones. They were instructed to pay attention to the stimulus stream and press a button when they heard the high-pitched tone (infrequent target stimulus) while disregarding the low-pitched tone (standard stimulus). The peak amplitude between 300–600 ms at electrode Pz was extracted and analyzed for oddball stimuli.

The N170 wave was based on a modified procedure we previously developed, where details of recording and analyses are provided [35, 36]. Briefly, in separate blocks, participants viewed either faces or buildings. For faces [37], participants had to determine the emotion displayed (afraid, angry, ashamed, happy, sad, or surprised); for buildings, they had to determine if they were 1 or 2 stories tall. The EEG was re-referenced to the grand average of the 19 electrodes. The mean amplitude between 140–180 ms was averaged across electrodes P7 and P8 for faces and buildings.

### Electrical stimulation

As described in detail previously [17], the tDCS was delivered by an Activa Dose II Iontophoresis Delivery Unit (IOMED, Inc), using a pair of saline-soaked sponge electrodes held in place by elastic bands. The 5 x 7 cm active electrode (anodal or cathodal) was placed over the left DLPFC (F3 EEG site), with the 5 x7 cm reference electrode over the right supraorbital cortex (Fp2 EEG site), in accordance with the 10/20 International System [38]. The current intensity was set at 2 mA with a current density of 0.057 mA/cm2 at the skin at each electrode. A variable resistor was included in series with each active electrode, which allowed for continuous monitoring of current delivered and ensured that equivalent intensity balanced at 1 mA per active electrode. In the sham condition, the current was turned on for 30 seconds and then ramped down to 0 mA. In this way, the participants experience the same initial sensation of mild tingling, thus preserving the sham manipulation [15]. The research assistants administering the procedure were not blinded to the stimulation condition.

### Procedures

We used a cross-over counter-balanced design. Participants came for three visits, approximately one week apart. The duration of the first visit was approximately 4 hours, whereas the second and third visits each took approximately 2.5 hours. At each visit they were administered one of three randomly assigned tDCS conditions: anodal over left DLPFC (cathodal over right supraorbital), cathodal over left DLPFC (anodal over right supraorbital), or sham. (During the first visit, they also completed the diagnostic interviews and questionnaires.) At each visit, participants received two 20-minute stimulation sessions, with approximately 100 minutes between the sessions, in which one stimulation was followed by cognitive testing and the other was followed by EEG. To address potential practice effects, the design of the study was fully counterbalanced, making practice effects orthogonal to the condition effects and thus avoiding confounds with the experimental conditions. Specifically, the order of assessments (cognitive and EEG) was counter-balanced across subjects. Further, within the cognitive assessments, we counter-balanced social cognitive and neurocognitive measures across subjects.

### Statistical analyses

Generalized Linear Mixed Models (GLMM) analyses were conducted, using IBM SPSS Statistics, Version 25, to examine the effects of tDCS on patients’ performance on social cognitive, neurocognitive, and EEG measures. For all non-significant findings, the results of post-hoc power analyses are presented as the critical N, that is, the sample size required for 80% power to detect an effect of this size. In cases where the estimated non-centrality parameter is negative (i.e., the observed differences between means are actually smaller than would be expected by chance), the critical N is reported as NA and observed power as 0, as the pattern of results is more consistent with the null hypothesis than any possible alternative; and regardless of how large the sample is, this pattern of results will never yield a significant result. The dataset is available in supporting information (see S1 Dataset).

## Results

GLMM analyses were conducted to examine the effects of tDCS on patients’ performance. tDCS condition (anodal, cathodal, and sham) was modeled as a within-subject effect, and a random effect for patient effects was included to account for individual differences between participants. As can be seen in Table 2, none of the social cognitive measures were significantly affected by the stimulation condition. Similarly, tDCS condition had no significant effect on nonsocial cognition, or the cognitive composite. The only measure significantly affected by stimulation was working memory, showing higher performance in the sham condition than in the anodal and cathodal conditions. Finally, none of the EEG measures demonstrated a significant effect of tDCS (see Table 2).

**Table 2:** Performance data on measures of social cognition, neurocognition, and EEG (N = 37).

	Anodal	Cathodal	Sham	F	p	f^2^	Observed	Critical
	M±SE	M±SE	M±SE				Power	N
Social Cognition								
MSCEIT	36.4±2.06	37.7±2.05	37.1±2.05	0.282	0.755	0.008	0	NA
EIT	44.9±1.39	43.9±1.37	44.9±1.39	0.475	0.624	0.013	0	NA
TASIT	22.1±0.71	22.4±0.70	22.5±0.70	0.457	0.635	0.013	0	NA
EAT	0.49±0.04	0.49±0.04	0.49±0.04	0.002	0.998	<0.001	0	NA
Neurocognition								
MCCB CCS	45.0±1.65	45.2±1.65	45.8.6±1.64	0.462	0.632	0.013	0	NA
MCCB SP	44.7±2.19	45.4±2.18	45.1±2.18	0.148	0.862	0.004	0	NA
MCCB WM	38.2±2.19	38.0±2.18	40.9±2.18	3.754	0.028	0.110		
MCCB VM	45.7±2.19	44.3±2.18	44.7±2.18	0.417	0.660	0.012	0	NA
MCCB RPS	51.4±1.68	53.1±1.67	52.5±1.67	0.683	0.509	0.020	0	NA
EEG								
MMN FZ	-3.44±0.30	-3.64±0.29	-3.35±0.29	0.529	0.592	0.015	0	NA
P300 Peak								
Latency	472.6±16.1	440.5±15.5	422.8±15.7	3.076	0.053	0.090	0.61	54
P300 Peak								
Amplitude	7.22±0.98	7.03±0.96	7.73±0.97	0.494	0.613	0.014	0	NA
N170 Emotion	-2.52±0.40	-3.26±0.39	-2.71±0.39	1.935	0.154	0.057	0.29	135
N170 Building	0.56±0.29	0.62±0.28	0.56±0.28	0.027	0.974	<0.001	0	NA

MSCEIT: Mayer-Salovey-Caruso Emotional Intelligence Test; EIT: Emotion Identification Test; TASIT: The Awareness of Social Inference Test; EAT: Emphatic Accuracy Task; MCCB: MATRICS Consensus Cognitive Battery; CCS: Cognitive Composite Score; SP: Speed of Processing; WM: Working Memory; VM: Verbal Memory; RPS: Reasoning and Problem Solving; EEG: Electroencephalogram; MMN: Mismatch Negativity; M±SE: Mean±Standard Error; Critical N is the sample size required for 80% power to detect the reported effect; In cases where the estimated non-centrality parameter is negative, the critical N is reported as NA and observed power as < 0.05.

GLMM analyses were also conducted to examine the common side effects of tDCS. As can be seen in Table 3, participants reported significantly more itching, pain, burning, and heat sensations following the anodal and cathodal stimulations, as compared with sham. However, none of the side effects presented substantial discomfort for the participants, as most of the symptoms were rated either as none or mild (below 1) on the scale, and none requested to discontinue the procedure.

**Table 3:** Side effects following tDCS administration (N = 37).

	Anodal	Cathodal	Sham	F	p
	M±SE	M±SE	M±SE		
Itching	1.27±0.19	1.41±0.18	0.50±0.18	8.801	<0.001
Pain	0.26±0.10	0.44±0.10	0.13±0.10	4.647	0.013
Burning	0.90±0.18	1.16±0.17	0.44±0.17	7.418	0.001
Heat	0.73±0.12	0.41±0.12	0.29±0.12	6.860	0.002
Pinching	0.32±0.12	0.39±0.12	0.19±0.12	0.836	0.438
Iron Taste	0.12±0.06	0.08±0.06	0.06±0.06	0.293	0.747
Fatigue	0.58±0.15	0.61±0.15	0.36±0.15	1.108	0.336

M±SE: Mean±Standard Error

## Discussion

In this study, we examined the effect of single tDCS administration in schizophrenia on a broad range of social cognitive, nonsocial neurocognitive, and ERP measures. Employing a commonly used montage in cognitive enhancement tDCS studies in a cross-over design, we found that none of the social cognitive measures nor any of the EEG measures were significantly affected by the stimulation condition. Similarly, tDCS condition had no significant effect on nonsocial neurocognition, including the cognitive composite score. The only measure significantly affected by stimulation was working memory, with both anodal and cathodal stimulations adversely affecting performance. The very small effect sizes, further validated by *post-hoc* power analyses (large Critical Ns), demonstrated that these findings were not due to lack of statistical power.

The present findings, combined with the inconsistency of therapeutic effects reported in studies of single tDCS administration, underscore the need to examine alternative tDCS montages. Along these lines, recent studies have examined the effects of tDCS at different intensities and/or across multiple sessions [39–42]. For example, Hoy et al. (2014) examined the effects of anodal left dorsolateral prefrontal tDCS at different intensities (1mA, 2mA, sham) and measured performance across three time points post-stimulation (0, 20 and 40 min). They found a significant improvement in performance over time following 2 mA stimulation only, demonstrating the importance of dose of stimulation [42]. Similarly, Brunelin et al. (2012) administered active 2 mA tDCS to individuals with schizophrenia for 20 minutes twice a day on 5 consecutive weekdays. They reported a robust reduction in auditory verbal hallucinations, with the beneficial effect lasting for up to 3 months [40]. Smith et al. (2015) conducted a randomized double-blind, sham-controlled study of the effects of 5 sessions of tDCS on cognitive and psychiatric symptoms in individuals with schizophrenia or schizoaffective disorder. They reported significant improvements after the fifth tDCS session in MCCB Composite score and on the MCCB Working Memory and Attention-Vigilance domain scores, with large effect sizes [41]. These latter two studies demonstrate the potentially cumulative effect of multiple tDCS session.

Another approach that should be incorporated in future studies involves online (task-concurrent) tDCS. In this montage, participants engage in cognitive training while concurrently receiving active tDCS. Recent studies suggest that this paradigm may be particularly effective for enhancing certain cognitive functions [43–45]. Au et al. (2016), for example, administered 7 days of working memory training concurrent with active either active (left or right DLPFC) or sham tDCS to a sample of healthy individuals. They found that active tDCS enhanced training performance, which was preserved several months after training completion [43]. Similarly, Ruff et al. (2017) examined the effect on healthy adults of three consecutive training sessions concurrent with tDCS on both spatial and verbal working memory. They compared active tDCS over DLPFC in a task-congruent (spatial-right, verbal-left), task-incongruent (spatial-left, verbal-right), and sham stimulation in regards to the efficacy of WM training and found a steeper learning curve when WM training was combined with task-congruent tDCS, with effects lasting for up to nine months and transferring to respective untrained tasks [44]. Finally, Oldrati et al. (2018) compared the effects of online vs. offline tDCS on enhancing training on a visuospatial task. They administered anodal tDCS of DLPFC either before or during training to a sample of healthy volunteers and found that, compared with the sham condition, the largest improvement occurred in the online tDCS condition [45]. These studies thus underscore the facilitative and long-lasting effects of tDCS when applied concurrently with cognitive training.

Considerable efforts have been made to find ways to enhance cognition in schizophrenia, mostly focusing on psychopharmacological approaches [4, 11, 46]. In recent years, neurostimulation has been developed as a non-invasive tool for cognitive enhancement [19, 21, 47, 48]. Neurostimulation has some advantages over psychopharmacology in that it is relatively safe with few side effects [49]. The tDCS procedure was well-tolerated in our study, with most common side effects being itching, pain, burning, and heat sensations, which followed both the anodal and cathodal stimulations. Importantly, most of the side effects were rated as mild or below, and none of the participants requested to discontinue the procedure. These findings are consistent with numerous reports [15, 20, 21], clearly establishing the safety and tolerability of single tDCS administration.

A number of limitations of the current study should be noted. In an effort to blind participants to the type of stimulation, we created a sham condition in which the current was turned on for 30 seconds and then ramped down to 0 mA. In this way, participants experienced the same initial sensation of mild tingling that is experienced during anodal and cathodal stimulations. Nonetheless, when comparing self-reported measures of side effects across the stimulation conditions, participants reported significantly more itching, pain, burning, and heat sensations following the anodal and cathodal stimulations, as compared with sham. Thus, although these side effects were very mild, findings suggest that actual tDCS conditions are experienced differently than sham. Additionally, the research assistants administering the procedure were not blinded to the stimulation condition, which could have also potentially influenced the sensations reported by the participants. Importantly, these findings are limited to effects of single tDCS administration, as assessed by changes from baseline to immediately post-stimulation. It could have been informative to conduct multiple tDCS sessions, while analyzing potential effects on a session to session basis. Unfortunately, practice effects, which are inherent in most performance-based measures, limit the applicability of these approaches in clinical trials of this type.

In summary, although these findings support the safety and tolerability of single tDCS administration, using a reasonably well-powered cross-over design, we failed to detect a differential tDCS effect of stimulation condition on neurocognitive and social cognitive performance-based tasks, as well as on EEG measures. These findings thus add to the growing body of evidence suggesting the potentially suboptimal dose of the single tDCS administration and point to the need for basic dose-finding studies in tDCS, as there would be for a new pharmacological intervention. Given its safety, tolerability, portability, relatively cheap cost, and ease of administration, once therapeutic dose is established, tDCS could potentially become a valuable therapeutic tool in the treatment of neurocognitive and social cognitive deficits in schizophrenia.

## Supporting information

S1 Checklist Checklist of items for reporting trials of Nonpharmacologic Treatments. (DOC)

S1 Protocol Approved protocol by the Institutional Review Board. (DOCX)

S1 Dataset Anonymized dataset. (XLSX)
