# Supplementary material for: Single transcranial direct current stimulation in schizophrenia: Randomized, cross-over study of neurocognition, social cognition, ERPs, and side effects
Source: PLoS One. 2018 May 7;13(5):e0197023. doi: 10.1371/journal.pone.0197023 (PMC5937783; doi:10.1371/journal.pone.0197023)
Supplement: S1 Protocol — (DOCX) [file pone.0197023.s002.docx]

**S1 IRB Protocol**

**1. LAY LANGUAGE SUMMARY:**

This study will examine the benefits of transcranial direct current stimulation (tDCS), a new tool that is being developed as a safe and non-invasive neurostimulation method, for improving neurocognitive and social cognitive functions in schizophrenia. This procedure is non-invasive and painless and it results in increase or decrease of spontaneous neuronal firing in the brain. Its safety and beneficial effect on cognition has been demonstrated in healthy individuals and several clinical populations. In this pilot study, we will examine the effect of tDCS on cognitive functions in 40 individuals with schizophrenia. Each participant will arrive for three visits, with approximately one week between each visit. During the first visit, participants will be interviewed about their psychiatric symptoms, personal life experiences, and emotional well being by a specially-trained interviewer. On each of the three visits, participants will receive one of three stimulations: a type of tDCS designed to increase neuronal firing, an alternative form of tDCS designed to decrease neuronal firing, and a sham tDCS (stimulation with no current). Immediately following the stimulation, participants will be asked to complete measures of mental abilities, including tests presented on a computer screen and paper-and-pencil tests. During each visit, participants will also undergo a standard measure of brain activity (EEG) while listening to tones. The first visit will last approximately five hours, and the other two visits will last approximately four hours each. The project will take approximately two years to complete.

| **PURPOSE OF THE STUDY, THE BACKGROUND AND THE LITERATURE REVIEW** |
| --- |

2. **Purpose of the Study:**

This study is an effort to pilot an innovative intervention for key cognitive deficits in schizophrenia. We propose to test the effects of unilateral administration of two types of transcranial direct current stimulations (tDCS) applied to frontal brain areas on neurocognitive and social cognitive functions, as well as on brainwave activity, in individuals with schizophrenia. The two types of tDCS are “anodal” and “cathodal” and they are designed to increase or decrease cortical activity, respectively, by modulating spontaneous neuronal network activity. We expect a differential effect of these types of stimulation on performance-based tasks and brainwave activity that are mediated by frontal brain regions.

**Specific Aims**

Given the exploratory nature of this pilot study, non-directional Aims are listed.

Specific Aim 1: To examine the effect of tDCS on neurocognitive functions (specifically, attention, speed of processing, verbal and visual learning, working memory, and reasoning and problem solving) in individuals with schizophrenia.

Specific Aim 2: To examine the effect of tDCS on social cognitive functions (specifically, emotional processing, affect perception, social perception, and theory of mind)in individuals with schizophrenia.

Specific Aim 3: To examine the effect of tDCS on EEG brain activity (using auditory oddball ERP) in individuals with schizophrenia.

3. **Background:**

**Cognition and Social Cognition in Schizophrenia**

Considerable evidence indicates that cognitive factors are key determinants of functional outcome for schizophrenia ^1-3^. These cognitive factors can be divided into neurocognition and social cognition. Neurocognition refers to a wide range of performance abilities in domains such as learning and memory, vigilance / attention, speed of processing, reasoning and problem solving, and working memory ^4, 5^. Cognitive deficits have been extensively documented in schizophrenia literature and are considered a *core feature* of the illness, meaning that these deficits represent a fundamental aspect of the illness, not simply the result of symptoms or the current treatments. Several lines of evidence converge to support this conclusion (summarized in ^6-13^).

For example, studies report that many patients demonstrate clear cognitive impairments before the onset of psychotic symptoms and other clinical features. Additionally, a subgroup of the first-degree relatives of schizophrenic patients who have no evidence of psychosis also demonstrate cognitive impairments of reduced severity, suggesting that certain cognitive deficits likely reflect genetic vulnerability to schizophrenia. On some measures of cognition, the level of impairment when patients are in psychotic episode is similar to that seen when their symptoms are under control or in full remission. Furthermore, the correlations between psychotic symptom severity and measures of cognitive performance are typically near zero. Findings are mixed for disorganized symptoms, and cognitive performance tends to be modestly related to negative symptoms. Both first- and second-generation antipsychotic medications have much stronger effects on psychotic symptoms as compared to cognition, suggesting that antipsychotic medications act on different neural systems from those that underlie cognitive impairments. Finally, schizophrenia has a modal pattern of cognitive deficits that is different from that seen in dementia, bipolar disorder, and depression ^14, 15^. The pattern and magnitude of cognitive deficits in schizoaffective disorder are typically not distinguishable from that of schizophrenia, and many studies combine data from both types of patients ^14^.

Social cognition refers to cognitive abilities that are needed to perceive, interpret, and process social information, and can be viewed as the ability to construct representations about others, oneself, and relations between others and oneself ^16-18^. Simply put, social cognition is people thinking about people. Studies of social cognition in schizophrenia have focused on several domains, including emotion perception, social context processing, attributional bias, and theory of mind (ToM) ^18^. These areas are consistently impaired in schizophrenia ^19^. Interest in social cognition was further stimulated by its inclusion as a key domain in the NIMH-sponsored MATRICS Consensus Cognitive Battery based on its relationship to outcome and potential role as a mediator between neurocognition and functional outcome ^4, 5^.Social cognitive deficits appear to be key determinants of daily functioning in schizophrenia, including instrumental activities, interpersonal functioning and vocational achievement ^20-24^. Disturbances in social cognition may be particularly germane to problems in forming and maintaining interpersonal relationships and interpersonal difficulties in work settings, problems common to many persons with schizophrenia. It is believed that disturbances in social cognition lead to social misperceptions that influence how an individual interprets and reacts to the behavior of others, which may consequently result in interpersonal difficulties and/or social withdrawal^16, 25^.

**A Novel Intervention to Improve Cognitive and Social Cognitive Deficits in Schizophrenia: Transcranial Direct Current Stimulation**

Considerable efforts have been made in recent years to stimulate the development of cognition-enhancing drugs including National Institute of Mental Health (NIMH) initiatives such as Measurement and Treatment Research to Improve Cognition in Schizophrenia (MATRICS), Treatment Units for Research on Neurocognition and Schizophrenia (TURNS), and Cognitive Neuroscience Treatment Research to Improve Cognition in Schizophrenia (CNTRICS) ^4, 14, 26-28^. The PI has had leadership roles on all these initiatives. Neurostimulation has advantages over pharmacological therapy in reducing many potentially dangerous systemic side effects. For example, vagus nerve stimulation is a widely accepted alternative to drug therapy in refractory epilepsy, and although showing much promise, it carries the risks associated with invasive surgery such as infection and hemorrhage^29, 30^. Currently transcranial direct current stimulation (tDCS) has been developed as non-invasive tool using neurostimulation to treat epilepsy^31^.

Unlike other non-invasive brain stimulation techniques such as transcranial electrical stimulation or transcranial magnetic stimulation, tDCS does not induce neuronal firing by suprathreshold neuronal membrane depolarization but rather modulates spontaneous neuronal network activity^32, 33^. At the neuronal level, the primary mechanism of action is a tDCS polarity-dependent shift (polarization) of resting membrane potential. Studies suggest that anodal-tDCS, or flow from anode to cathode, generally enhances cortical activity and excitability, whereas cathodal-tDCS, or flow from the cathode to anode, has the opposite effect^34, 35^. Animal studies have shown that changes in excitability are reflected in both spontaneous firing rates^36^, and responsiveness to afferent synapticinputs^37, 38^. It is this primary polarization mechanism that underlies the acute effects of low-intensity DC currents on cortical excitability in humans^39^.

Animal and human studies have now demonstrated the safety and potential clinical benefits of tDCS. This procedure has been used in various research laboratories world-wide for the last 30-40 years and has been investigated in numerous neurologic and psychiatric diagnoses ^29-31, 40-47^. In a review of 567 tDCS sessions administered over cortical areas on a total of 102 healthy participants and patients, none requested the stimulation to be terminated or needed any medical intervention during or after tDCS administration ^45^.

In healthy volunteers, tDCS studies have mostly focused on cognitive enhancement. For example, Anodal tDCS over the dorsolateral prefrontal cortex (DLPFC), compared with sham stimulation, significantly improved working-memory performance in healthy adults^48-50^.Nitsche et al. (2003) found that tDCS of the primary motor cortex facilitated implicit motor learning^51^. Other studies have demonstrated that stimulation of Broca’s and other language-related regions enhanced language performance, including acquisition of object names and novel grammar, faster object naming, and increased verbal fluency^52-55^. In a recent investigation, stimulation of the left DLPFC improved performance on the remote associations test (RAT), a measure that has been correlated with complex cognitive functions, such as creative thinking, executive function, and general intelligence^56^.

A number of studies have also shown an effect of tDCS on social cognition. For example, in one study, 36 healthy volunteers performed a gambling task while being administered either anodal over the right with cathodal over the left DLPFC, anodal over the left with cathodal over the right DLPFC, or sham tDCS^57^. The number of times participants choose the most likely outcome and the decision times were calculated and averaged. As predicted by the investigators, during the right anodal/left cathodal stimulation, participants tended to select the safer alternative compared with the other groups, which was interpreted by the investigators to suggest decreased risk-taking behavior.

tDCS has also been evaluated in several clinical populations. In the first study with tDCS in adult epilepsy patients, Fregni et al. (2006) randomized patients with medically-refractory focal and generalized epilepsy to double-blinded, sham-controlled tDCS treatment. They noted a reduction in the number epileptiform discharges on EEG, as well as a reduction in the number of clinical seizures, and also found it to be safe with no accounts of seizure induction^43^. In another study, anodal tDCS to the left frontal lobe resulted in improvements in naming accuracy in patients with left hemisphere strokes and chronic aphasia^40^. Brunoni et al (2011) explored the effectiveness of tDCS in the treatment of unipolar and bipolar depressive disorder. They applied tDCS over the DLPFC (anodal electrode on the left and cathodal on the right) twice-daily, for 5 consecutive days. The authors reported thatall patients tolerated treatment well without adverse effects, and after the fifth tDCS session, depressive symptoms in both study groups diminished, with beneficial effects persisting at one week and one month^42^.

Pertinent to the current proposal, Brunelin (2012) recently reported preliminary data showing that bifocal stimulation (anodal over DLPFC and cathodal over tempo-parietal cortex) improved scores on measures of auditory hallucinations and overall positive and negative symptoms in 2 patients with refractory schizophrenia. The clinical efficacy appeared immediately after stimulation sessions and was maintained or continued to improve during at least 3 months. The author noted that no adverse events were reported, and that the two patients only described a transient mild tingling or a slight itching sensation associated with the onset of stimulation, thereby confirming the safety of this technique ^58^. Additionally, Vercammen et al. (2011) examined whether anodal tDCS to the left DLPFC would reverse probabilistic association learning deficits in 20 individuals with schizophrenia. The authors reported no adverse effects following the procedure and demonstrated a beneficial effect of tDCS on performance, as assessed by the weather prediction test, in a subsets of individuals ^46^. Mattai et al. (2011) investigated the tolerability aspects of tDCS in the childhood-onset schizophrenia (COS) population. The investigators administered bilateral anodal DLPFC stimulation or bilateral cathodal superior temporal gyrus (STG) stimulation to children between the ages 10-17, demonstrating the safety and tolerability of this procedure in a pediatric population ^59^. Recently, Hoy et al. (2014) examined the effects of anodal left dorsolateral prefrontal tDCS at different intensities (1mA, 2mA, sham) and measured performance across three time points post-stimulation (0, 20 and 40 min) ^60^. They found a significant improvement in performance over time following 2 mA stimulation only, demonstrating the feasibility of tDCS for enhancing cognitive performance in schizophrenia, as well as the importance of dose of stimulation.

**Significance and future directions**

Individuals with schizophrenia experience very high levels of disability and poor community outcome, resulting in a major public health concern. We propose to pilot an innovative intervention technique to improve key determinants of functional outcome, namely neurocognition and social cognition. This pilot study will provide the groundwork and preliminary data for a grant application for conducting a larger-scale clinical trial on the efficacy of this intervention. As described in detail above, this procedure is non-invasive and painless and it results in increase or decrease of spontaneous neuronal firing in the brain. Its safety and beneficial effect on cognition has been demonstrated in healthy individuals and several clinical populations. Thus, given its safety, tolerability, portability, relatively cheap cost, and ease of administration, tDCS could potentially be a valuable therapeutic tool in the treatment of neurocognitive and social cognitive deficits in schizophrenia, leading to improved quality of life for these individuals.

| **CHARACTERISTICS OF THE SUBJECT POPULATION** |
| --- |

**Inclusion/Exclusion Criteria:**

*What are the criteria for inclusion?*

All patients will receive a diagnostic interview with the Structured Clinical Interview for DSM-IV and must meet the following criteria:

a. DSM-IV Diagnosis of Schizophrenia or Schizoaffective Disorder

b. Age 18 - 55

c. Able to understand spoken English sufficiently to comprehend testing procedures

d. Estimated premorbid IQ > 70 (based on reading ability)

e. Must be under the current ongoing care of a Psychiatrist, Psychologist, or other qualified mental health professional

*What are the criteria for exclusion?*

Potential patient subjects will be excluded if they do not meet the following criteria:

a. No metal in cranium, cardiac lines/pacemaker, medication pump, or increased intracranial pressure

b. No lifetime history of serious head injury (LOC > 1 hr.)

c. No sedatives or Benzodiazepines within 12 hours of testing

d. No clinically significant neurological disease (e.g., seizures)

e. No history of mental retardation or developmental disability

f. No Alcohol or substance dependence in the last 6 months

g. No Alcohol or substance abuse in the last 1 month

h. No change in medication in past 6 weeks

i. No change in in living situation in the last 2 months

j. No inpatient hospitalization in past 3 months

k. No participation in prior studies involving tDCS

| **METHODS AND PROCEDURES** |
| --- |

**Methods and Procedures Applied to Human Subjects:**

We plan to recruit 40 individuals diagnosed with Schizophrenia. Participants will range from 18–55 years old. We will select stable, medicated patients who have not been hospitalized nor had any changes in their antipsychotic medications during the previous two months. Subjects will not be tested within 12 hours of taking medications that may affect cognition (such as benzodiazepines and anticholinergics). All medications will be recorded. We expect most of the patients to be relatively chronic (mean illness chronicity approximately 15 years, based on previous studies), although we will not select for chronicity.

All participants will provide written informed consent after receiving a full explanation of the procedures according to approvals by the Institutional Review Boards. They will arrive for three visits. In the first visit, they will be consented, complete the diagnostic interview, the rating scales, and will undergo one of three randomly assigned stimulation conditions: 1) anodal tDCS, 2) cathodalt DCS, and 3) sham stimulation. They will also undergo an EEG procedure, in order to more directly assess the effect of tDCS on brain function. This visit will take approximately five hours to complete, and breaks will be given as needed. Immediately following stimulation, participants will complete neurocognitive, social cognitive, and EEG measures. There will be two 20-minute stimulation sessions, with a break of one hour between the sessions. After each session, subjects will be asked to complete a portion of the basic cognitive, social cognitive, and EEG measures, so that half of the measures will be completed after the first 20 minute stimulation session and the other half will be completed after the second 20 minute stimulation session. The order of administration of the neurocognitive and social cognitive measures will be randomized across participants within each group, while the EEG measure will be administered last. During the second and third visits, participants will undergo the remaining two stimulation conditions, one randomly assigned condition each visit. These visits will take approximately four hours to complete, and each visit will be separated by at least one week from the previous visit.

The measures and procedures are described in detail below.

**A. INTERVIEW-BASED ASSESSMENTS**

All patients will be interviewed using the Psychiatric and Social History Schedule, which is a brief semi-structured interview that includes questions about occupational and educational history, current residence, past hospitalizations (if any), and current medications (if any). In addition, all subjects will be screened with the Wechsler Test of Adult Reading (WTAR) to briefly asses their IQ through reading ability.

Symptom ratings will include the Expanded Brief Psychiatric Rating Scale and the Scale for the Assessment of Negative Symptoms.

*Expanded Brief Psychiatric Rating Scale* (BPRS ^61^). The Expanded BPRS will be administered by raters who have been extensively trained to reliability. The training methods have been described in detail and include a training component as well as ongoing quality assurance ^61^. The BPRS will cover a two-week period and will yield clusters for Thinking Disturbance (positive symptom), Withdrawal / Retardation (negative symptom) as well as a total score and items relevant to disorganized symptoms. The expanded version of this scale is based on a semi-structured interview with specific anchor points.

*Scale for the Assessment of Negative Symptoms* (SANS ^62^). This scale includes five areas of negative symptoms including affective flattening, alogia, avolition-apathy, anhedonia-asociality, and attention. This is also given as an interview, usually in conjunction with the BPRS. The training procedure for the SANS is similar to the one for the BPRS.

**B. NEUROCOGNITIVE AND SOCIAL COGNITIVE MEASURES**

1. *Neurocognition*: Neurocognitive functions will be assessed using the MATRICS Consensus Cognitive Battery (MCCB). This battery is one of the key deliverables of the MATRICS Initiative and it has gone through extensive review and a detailed selection process ^4, 63, 64^. The PI of this application was the Co-chair of the MATRICS Neurocognition Committee, which had the responsibility of overseeing the selection of the MCCB. The tests were selected from over 90 nominated tests and assess the primary cognitive domains found to be impaired in schizophrenia, such as attention/vigilance, speed of processing, verbal and visual learning, working memory, and reasoning and problem solving. The specific tests will be randomized across subjects and the total time of administration is approximately 60 minutes.

2. *Social cognition*: Standardized social cognition assessments will include a number of primary measures of social cognition that have been used in schizophrenia research^18^. *Managing Emotions component of Mayer-Salovey-Caruso Emotional Intelligence Test (MSCEIT)*^65^consists of 141 items and 8 ability subtests, which assess four components (branches) of emotional processing. For this application, we will focus on the *Managing Emotions* component and will administer branches 1 and 4 only. *Emotion Identification Test from Ekman Program* is a test of identification of facial emotion based on photographs from the software developed by Ekman ^66^. *Profile of Nonverbal Sensitivity (PONS)* is used to assess social perception^67, 68^. Scenes in this videotape-based measure last two seconds and contain facial expressions, voice intonations, and/or bodily gestures of a Caucasian female. *The Awareness of Social Inference Test (TASIT)*^69^ is a videotape test of theory of mind in various social contexts. The specific measures will be randomized across subjects and the total time of administration is approximately 40 minutes.

### C. EEG MEASURES

To assess the effect of tDCS on brain function, participants will also undergo an auditory oddball ERP protocol that has been well standardized in our lab, at baseline and following tDCS administration. Participants will be presented with standard and duration-deviant tones binaurally using foam ear inserts (1 kHz 85 dB sound pressure level, with 10 ms rise/fall) with a fixed stimulus onset asynchrony of 500 ms, using E-Prime 2.0. Standard (90% probability; 50 ms duration) and deviant (10% probability; 100 ms duration) tones will be presented in a fixed, pseudorandom order (with the restriction that at least 4 standard tones were presented between deviant tones). Two-thousand total trials will be administered. During the 20-minute EEG recording, subjects will be instructed to watch a silent movie to divert attention from the stimuli. This procedure will always be administered last. Total administration time, including preparation, is 45 minutes.

EEG recordings will be acquired with a 64-channel BioSemiActiveTwo amplifier (Biosemi B. V., Amsterdam, Netherlands). Data will be sampled at 1024 Hz with a bandpass of 0.16 to 100 Hz. Additional electrodes will be placed above and below the left eye and at the outer canthi of both eyes to monitor blinks and eye movements. Each active electrode will be measured online with respect to a common mode sense electrode during data collection, forming a monopolar channel. An additional electrode will be placed at the nose tip and all EEG data were re-referenced offline to this electrode.

Data processing will be performed offline using BrainVision Analyzer 2 software (Brain Products, Gilching, Germany). The MMN and P3a will be derived from electrodes at frontocentral sites, based on previous studies (AF3, AF4, AF7, AF8, AFz, Cz, F1, F2, F3, F4, F5, F6, F7, F8, FC1, FC2, FC3, FC4, FC5, FC6, FCz, Fp1, Fp2, Fpz, Fz). Epochs that contained activity exceeding ± 75 μV at these electrode sites will be automatically rejected. MMN and P3a waveforms will be generated by subtracting standard from deviant averaged waveforms. The waveforms will then be low-pass filtered at 20 Hz (zerophase shift, 24 dB/octave rolloff) to remove any residual high-frequency artifact. MMN and P3a amplitudes will be measured as the mean of activity in the 135-205 ms and 250-350 ms latency range, respectively.

### D. TDCS PROCEDURE

Based on the recommended montage for left DLPFC stimulation ^60, 70^, one electrode will be placed over the left DLPFC (F3) and the other over the right supraorbital region. Participants will receive one of the three randomly assigned tDCS conditions per visit: anodal tDCS over left DLPFC (cathodal over right supraorbital), cathodal tDCS over left DLPFC (anodal over right supraorbital), and sham stimulation. The tDCS will be delivered by anActiva Dose II Iontophoresis Delivery Unit, FDA-approved iontophoresis device, using a pair of saline-soaked sponge electrodes held in place by elastic bands. The two electrodes are 5 x 7 cm, and the current intensity will be set at 0.08 mA/cm^2^ (2 mA) at the skin and will be maintained for two 20 minute sessions with a one hour break between. The current intensity and duration is comparable to those used in many previous studies ^44, 54, 71^. In the sham condition, the current will be turned on for 30 seconds and then ramped down to 0 mA. In this way, the participants experience the same initial sensation of mild tingling as the cathodal-tDCS group, thus preserving the sham manipulation ^45^.

Immediately following each of the two tDCS administrations, participants will complete the neurocognitive, social cognitive, and EEG assessments, as described above. After completion of the procedures, we will interview the subject informally for any adverse unexpected reactions that may have taken place during the study and will request that the subject describe any sensations or feelings experienced during or after the stimulation.

**FDA Approval:**

The direct current stimulation is delivered by an Activa Dose II Iontophoresis Delivery Unit, an FDA approved iontophoresis device. The Activa Dose II Iontophoresis Delivery Unit is a substantively equivalent version of the IomedPhoresor II Iontophoretic Drug Delivery System, which is FDA-approved for the administration of soluble salts and other drugs, including local dermal anesthesia, into the body for medical purposes as an alternative to hypodermic injection. We will use the Activa Dose II Iontophoresis Delivery Unit for transcranial direct current stimulation (tDCS) over the cortex for the purpose of modulating spontaneous neuronal firing. This practice represents a research use that is an off-label use of an FDA approved device. However this device has been utilized in the literature as a tDCS device intended in our study ^44, 54, 71^. Overall, tDCS is very well-tolerated. The most common side effect of tDCS is described as a mild tingling at the electrode site. However, this sensation is only present during the first few seconds of stimulation ^51, 54^. While potential risks of tingling and burning exist, we do not exceed conservative estimated limits of current density, even for children, and our limits are consistent with prior studies utilizing extracephalic electrodes. In our study protocol, we extrapolate research with tDCS that shows direct current polarization non-invasively and painlessly results in hyperpolarization or depolarization of the cerebral cortex, depending on current flow. Seizure induction risk is exceedingly low and has never been reported with tDCS in humans or animals ^31, 72^. Therefore, use of tDCS in this population represents non-significant risk by FDA criteria.

Note that the policy of FDA with regard to investigations that employ transcranial applications of magnetic stimulators is that in any study, regardless of the device, the population being studied, or the stimulation parameters used, the responsibility for determining whether an investigation poses a significant risk to subjects falls on the investigator's IRB. The FDA acknowledges and supports the authority of IRBs to make these decisions.

The Activa Dose II Iontophoresis Delivery Unit will be loaned to us by our consultant, Allan Wu, MD, an Assistant Professor in the UCLA Department of Neurology who is an expert in tDCS. He will provide all training and safety certification for the research staff who will administer the tDCS procedures. There will be annual review and training in the administration and safety procedures associated with use of the device and tDCS procedures.

**References**

**1.** Green MF. What are the functional consequences of neurocognitive deficits in schizophrenia? *American Journal of Psychiatry.* 1996;153(3):321-330.

**2.** Green MF, Kern RS, Braff DL, Mintz J. Neurocognitive deficits and functional outcome in schizophrenia: Are we measuring the "right stuff"? *Schizophrenia Bulletin.* 2000;26:119-136.

**3.** Green MF, Kern RS, Heaton RK. Longitudinal studies of cognition and functional outcome in schizophrenia: implications for MATRICS. *Schizophrenia Research.* 2004;72:41-51.

**4.** Green MF, Nuechterlein KH, Gold JM, et al. Approaching a consensus cognitive battery for clinical trials in schizophrenia: The NIMH-MATRICS conference to select cognitive domains and test criteria. *Biological Psychiatry.* 2004;56:301-307.

**5.** Nuechterlein KH, Barch DM, Gold JM, Goldberg TE, Green MF, Heaton RK. Identification of separable cognitive factors in schizophrenia. *Schizophrenia Research.* 2004;72:29-39.

**6.** Nuechterlein KH, Dawson ME, Green MF. Information-processing abnormalities as neuropsychological vulnerability indicators for schizophrenia. *Acta Psychiatrica Scandinavica.* 1994;90(Suppl. 384):71-79.

**7.** Goldberg TE, Green MF. Neurocognitive functioning in patients with schizophrenia: An overview. In: Davis KL, Charney D, Coyle JT, Nemeroff C, eds. *Neuropsychopharmacology: The Fifth Generation of Progress*. Philadelphia: Lippincott, Williams & Wilkins; 2002.

**8.** Nuechterlein KH, Dawson ME. Information processing and attentional functioning in the developmental course of schizophrenia disorders. *Schizophrenia Bulletin.* 1984;10:160-203.

**9.** Braff D. Information processing and attention dysfunctions in schizophrenia. *Schizophrenia Bulletin.* 1993;19:233-259.

**10.** Gold JM. Cognitive deficits as treatment targets in schizophrenia. *Schizophrenia Research.* 2004;72:21-28.

**11.** Cornblatt B, Lenzenweger MF, Dworkin R, Erlenmeyer-Kimling L. Childhood attentional dysfunction predicts social deficits in unaffected adults at risk for schizophrenia. *British Journal of Psychiatry.* 1992;161(Suppl. 18):59-64.

**12.** Harvey PD, Keefe RSE. Studies of the cognitive change in patients with schizophrenia following novel antipsychotic treatment. *American Journal of Psychiatry.* 2001;158:176-184.

**13.** Gold JM, Green MF. Neurocognition in schizophrenia. In: Sadock BJ, Sadock VA, eds. *Comprehensive Textbook of Psychiatry*. 8th ed. Baltimore: Lippincott, Williams & Wilkins; 2004:1436-1448.

**14.** Buchanan RW, Davis M, Goff D, et al. A summary of the FDA-NIMH-MATRICS workshop on clinical trial design for neurocognitive drugs for schizophrenia. *Schizophrenia Bulletin.* 2005;31:5-19.

**15.** Heinrichs RW, Zakzanis KK. Neurocognitive deficit in schizophrenia: A quantitative review of the evidence. *Neuropsychology.* 1998;12:426-445.

**16.** Penn DL, Corrigan PW, Bentall RP, Racenstein JM, Newman L. Social cognition in schizophrenia. *Psychological Bulletin.* 1997;121:114-132.

**17.** Pinkham AE, Penn DL, Perkins DO, Lieberman JA. Implications of a neural basis for social cognition for the study of schizophrenia. *American Journal of Psychiatry.* 2003;160(5):815-824.

**18.** Green MF, Olivier B, Crawley JN, Penn DL, Silverstein S. Social cognition in schizophrenia: Recommendations from the MATRICS New Approaches Conference. *Schizophrenia bulletin.* 2005;31:882-887.

**19.** Edwards J, Jackson HJ, Pattison PE. Emotion recognition via facial expression and affective prosody in schizophrenia: A methodological review. *Clinical Psychology Review.* 2002;22:789-832.

**20.** Kee KS, Green MF, Mintz J, Brekke JS. Is emotional processing a predictor of functional outcome in schizophrenia? *Schizophrenia Bulletin.* 2003;29:487-497.

**21.** Corrigan PW, Toomey R. Interpersonal problem solving and information processing in schizophrenia. *Schizophrenia Bulletin.* 1995;21:395-403.

**22.** Mueser KT, Doonan B, Penn DL, et al. Emotion recognition and social competence in chronic schizophrenia. *Journal of Abnormal Psychology.* 1996;105:271-275.

**23.** Penn DL, Spaulding WD, Reed D, Sullivan M. The relationship of social cognition to ward behavior in chronic schizophrenia. *Schizophrenia Research.* 1996;20:327-335.

**24.** Couture SM, Penn DL, Roberts DL. The Functional Significance of Social Cognition in Schizophrenia: A Review. *Schizophrenia Bulletin.* October 1, 2006 2006;32(suppl 1):S44-63.

**25.** Green MF, Nuechterlein KH. Should schizophrenia be treated as a neurocognitive disorder? *Schizophrenia Bulletin.* 1999;25:309-319.

**26.** Marder SR, Fenton WS. Measurement and treatment research to improve cognition in schizophrenia: NIMH MATRICS Initiative to support the development of agents for improving cognition in schizophrenia. *Schizophrenia Research.* 2004;72:5-10.

**27.** Carter CS, Barch DM. Cognitive neuroscience-based approaches to measuring and improving treatment effects on cognition in schizophrenia: the CNTRICS initiative. *Schizophrenia bulletin.* 2007;33(5):1131-1137.

**28.** Stover EL, Brady L, Marder SR. New paradigms for treatment development. *Schizophrenia bulletin.* 2007;33(5):1093-1099.

**29.** Jobst BC. Electrical Stimulation in Epilepsy: Vagus Nerve and Brain Stimulation. *Current Treatment Options in Neurology.* Sep;12(5):443-453.

**30.** Jobst BC, Darcey TM, Thadani VM, Roberts DW. Brain stimulation for the treatment of epilepsy. *Epilepsia.*51:88-92.

**31.** Nitsche MA, Paulus W. Noninvasive Brain Stimulation Protocols in the Treatment of Epilepsy: Current State and Perspectives. *Neurotherapeutics.* Apr 2009;6(2):244-250.

**32.** Priori A, Hallett M, Rothwell JC. Repetitive transcranial magnetic stimulation or transcranial direct current stimulation? *Brain Stimulation.* Oct 2009;2(4):241-245.

**33.** Zaghi S, Acar M, Hultgren B, Boggio PS, Fregni F. Noninvasive Brain Stimulation with Low-Intensity Electrical Currents: Putative Mechanisms of Action for Direct and Alternating Current Stimulation. *Neuroscientist.* Jun;16(3):285-307.

**34.** Bindman LJ, Redfearn JW, Lippold OCJ. RELATION BETWEEN SIZE + FORM OF POTENTIALS EVOKED BY SENSORY STIMULATION + BACKGROUND ELECTRICAL ACTIVITY IN CEREBRAL CORTEX OF RAT. *Journal of Physiology-London.* 1964;171(1):1-&.

**35.** Nitsche MA, Paulus W. Excitability changes induced in the human motor cortex by weak transcranial direct current stimulation. *Journal of Physiology-London.* Sep 2000;527(3):633-639.

**36.** Creutzfeldt O, Fromm GH, Kapp H. INFLUENCE OF TRANSCORTICAL D-C CURRENTS ON CORTICAL NEURONAL ACTIVITY. *Experimental Neurology.* 1962;5(6):436-&.

**37.** Bikson M, Inoue M, Akiyama H, et al. Effects of uniform extracellular DC electric fields on excitability in rat hippocampal slices in vitro. *Journal of Physiology-London.* May 2004;557(1):175-190.

**38.** Jefferys JGR. INFLUENCE OF ELECTRIC-FIELDS ON THE EXCITABILITY OF GRANULE CELLS IN GUINEA-PIG HIPPOCAMPAL SLICES. *Journal of Physiology-London.* 1981;319(OCT):143-152.

**39.** Priori A, Berardelli A, Rona S, Accornero N, Manfredi M. Polarization of the human motor cortex through the scalp. *Neuroreport.* Jul 1998;9(10):2257-2260.

**40.** Baker JM, Rorden C, Fridriksson J. Using Transcranial Direct-Current Stimulation to Treat Stroke Patients With Aphasia. *Stroke.* Jun;41(6):1229-1236.

**41.** Brunelin. Efficacy and safety of bifocal tDCS as an interventional treatment for refractory schizophrenia. *Brain Stimulation.* in press.

**42.** Brunoni AR, Ferrucci R, Bortolomasi M, et al. Transcranial direct current stimulation (tDCS) in unipolar vs. bipolar depressive disorder. *Progress in Neuro-Psychopharmacology & Biological Psychiatry.* Jan;35(1):96-101.

**43.** Fregni F, Thome-Souza S, Nitsche MA, Freedman SD, Valente KD, Pascual-Leone A. A controlled clinical trial of cathodal DC polarization in patients with refractory epilepsy. *Epilepsia.* Feb 2006;47(2):335-342.

**44.** Mattai A, Miller R, Weisinger B, et al. Tolerability of transcranial direct current stimulation in childhood-onset schizophrenia. *Brain Stimulation.* in press.

**45.** Poreisz C, Boros K, Antal A, Paulus W. Safety aspects of transcranial direct current stimulation concerning healthy subjects and patients. *Brain Research Bulletin.* May 2007;72(4-6):208-214.

**46.** Vercammen A, Rushby JA, Loo C, Short B, Weickert CS, Weickert TW. Transcranial direct current stimulation influences probabilistic association learning in schizophrenia. *Schizophrenia Research.* Sep;131(1-3):198-205.

**47.** Nitsche MA, Liebetanz D, Lang N, Antal A, Tergau F, Paulus W. Safety criteria for transcranial direct current stimulation (tDCS) in humans. *Clinical Neurophysiology.* Nov 2003;114(11):2220-2222.

**48.** Fregni F, Boggio PS, Nitsche M, et al. Anodal transcranial direct current stimulation of prefrontal cortex enhances working memory. *Experimental Brain Research.* Sep 2005;166(1):23-30.

**49.** Ohn SH, Park CI, Yoo WK, et al. Time-dependent effect of transcranial direct current stimulation on the enhancement of working memory. *Neuroreport.* Jan 2008;19(1):43-47.

**50.** Zaehle T, Sandmann P, Thorne JD, Jancke L, Herrmann CS. Transcranial direct current stimulation of the prefrontal cortex modulates working memory performance: combined behavioural and electrophysiological evidence. *Bmc Neuroscience.* Jan;12.

**51.** Nitsche MA, Schauenburg A, Lang N, et al. Facilitation of implicit motor learning by weak transcranial direct current stimulation of the primary motor cortex in the human. *Journal of Cognitive Neuroscience.* May 2003;15(4):619-626.

**52.** de Vries MH, Barth ACR, Maiworm S, Knecht S, Zwitserlood P, Floel A. Electrical Stimulation of Broca's Area Enhances Implicit Learning of an Artificial Grammar. *Journal of Cognitive Neuroscience.* Nov;22(11):2427-2436.

**53.** Floel A, Rosser N, Miichka O, Knecht S, Breitenstein C. Noninvasive brain stimulation improves language learning. *Journal of Cognitive Neuroscience.* Aug 2008;20(8):1415-1422.

**54.** Iyer MB, Mattu U, Grafman J, Lomarev M, Sato S, Wassermann EM. Safety and cognitive effect of frontal DC brain polarization in healthy individuals. *Neurology.* Mar 2005;64(5):872-875.

**55.** Sparing R, Dafotakis M, Meister IG, Thirugnanasambandam N, Fink GR. Enhancing language performance with non-invasive brain stimulation - A transcranial direct current stimulation study in healthy humans. *Neuropsychologia.* 2008;46(1):261-268.

**56.** Cerruti C, Schlaug G. Anodal Transcranial Direct Current Stimulation of the Prefrontal Cortex Enhances Complex Verbal Associative Thought. *Journal of Cognitive Neuroscience.* Oct 2009;21(10):1980-1987.

**57.** Fecteau S, Knoch D, Fregni F, Sultani N, Boggio P, Pascual-Leone A. Diminishing risk-taking Behavior by modulating activity in the prefrontal cortex: A direct current stimulation study. *Journal of Neuroscience.* Nov 2007;27(46):12500-12505.

**58.** Brunelin J, Mondino M, Haesebaert F, Saoud M, Suaud-Chagny MF, Poulet E. Efficacy and safety of bifocal tDCS as an interventional treatment for refractory schizophrenia. *Brain Stimulation.* Jul 2012;5(3):431-432.

**59.** Mattai A, Miller R, Weisinger B, et al. Tolerability of transcranial direct current stimulation in childhood-onset schizophrenia. *Brain Stimulation.* Oct 2011;4(4):275-280.

**60.** Hoy KE, Arnold SL, Emonson MRL, Daskalakis ZJ, Fitzgerald PB. An investigation into the effects of tDCS dose on cognitive performance over time in patients with schizophrenia. *Schizophrenia Research.* May 2014;155(1-3):96-100.

**61.** Ventura J, Green MF, Shaner A, Liberman RP. Training and quality assurance with the brief psychiatric rating scale: 'The Drift Busters'. *International Journal of Methods in Psychiatric Research.* 1993;3:221-224.

**62.** Andreasen NC. *The scale for the assessment of negative symptoms (SANS).* Iowa City, IA: The University of Iowa; 1984.

**63.** Kern RS, Nuechterlein KH, Green MF, et al. The MATRICS Consensus Cognitive Battery: Part 2. co-norming and standardization. *American Journal of Psychiatry.* 2008;165:214-220.

**64.** Nuechterlein KH, Green MF, Kern RS, et al. The MATRICS Consensus Cognitive Battery: Part 1. test selection, reliability, and validity. *American Journal of Psychiatry.* 2008;165:203-213.

**65.** Mayer JD, Salovey P, Caruso DR. *Technical Manual for the MSCEIT (Version 2.0)*. Toronto, Canada: Multi-Health Systems; 2002.

**66.** *Subtle Expression Training Tool (SETT) & Micro Expression Training Tool (METT)* [computer program]. Version: Paul Ekman [www.paulekman.com.;](http://www.paulekman.com.;) 2004.

**67.** Ambady N, Hallahan M, Rosenthal R. On judging and being judged accurately in zero-acquaintance situations. *Journal of Personality and Social Psychology.* 1995;69:519-529.

**68.** Rosenthal R, Hall JA, DiMatteo MR, Rogers PL, Archer D. *Sensitivity to nonverbal communication: The PONS test.* Baltimore: Johns Hopkins University Press; 1979.

**69.** McDonald S, Flanagan S, Rollins J. *The Awareness of Social Inference Test*. Suffolk, England: Thames Valley Test Company Limited; 2002.

**70.** Nitsche MA, Cohen LG, Wassermann EM, et al. Transcranial direct current stimulation: State of the art 2008. *Brain Stimulation.* Jul 2008;1(3):206-223.

**71.** Koenigs M, Ukueberuwa D, Campion P, Grafman J, Wassermann E. Bilateral frontal transcranial direct current stimulation: Failure to replicate classic findings in healthy subjects. *Clinical Neurophysiology.* Jan 2009;120(1):80-84.

**72.** Liebetanz D, Klinker F, Hering D, et al. Anticonvulsant effects of transcranial direct-current stimulation (tDCS) in the rat cortical ramp model of focal epilepsy. *Epilepsia.* Jul 2006;47(7):1216-1224.
